# The Differential Response of Proteins to Macromolecular Crowding

**DOI:** 10.1371/journal.pcbi.1005040

**Published:** 2016-07-29

**Authors:** Michela Candotti, Modesto Orozco

**Affiliations:** 1 Institute for Research in Biomedicine (IRB Barcelona), The Barcelona Institute of Science and Technology, Barcelona, Spain; 2 Joint BSC-IRB Research Program in Computational Biology, Barcelona, Spain; 3 Department of Biochemistry and Molecular Biology, University of Barcelona, Barcelona, Spain; Baltimore, UNITED STATES

## Abstract

The habitat in which proteins exert their function contains up to 400 g/L of macromolecules, most of which are proteins. The repercussions of this dense environment on protein behavior are often overlooked or addressed using synthetic agents such as poly(ethylene glycol), whose ability to mimic protein crowders has not been demonstrated. Here we performed a comprehensive atomistic molecular dynamic analysis of the effect of protein crowders on the structure and dynamics of three proteins, namely an intrinsically disordered protein (ACTR), a molten globule conformation (NCBD), and a one-fold structure (IRF-3) protein. We found that crowding does not stabilize the native compact structure, and, in fact, often prevents structural collapse. Poly(ethylene glycol) PEG500 failed to reproduce many aspects of the physiologically-relevant protein crowders, thus indicating its unsuitability to mimic the cell interior. Instead, the impact of protein crowding on the structure and dynamics of a protein depends on its degree of disorder and results from two competing effects: the excluded volume, which favors compact states, and quinary interactions, which favor extended conformers. Such a viscous environment slows down protein flexibility and restricts the conformational landscape, often biasing it towards bioactive conformations but hindering biologically relevant protein-protein contacts. Overall, the protein crowders used here act as unspecific chaperons that modulate the protein conformational space, thus having relevant consequences for disordered proteins.

## Author Summary

Most in vitro and in silico biophysical experiments generally study proteins in an isolated environment, overlooking that their natural environment—the cell cytoplasm—is a solution that is highly populated by proteins. To address this knowledge gap, here we explored how a crowded environment alters the conformational sampling of three proteins, each with a different degree of disorder and flexibility. We simulated a crowded system composed by the three proteins and reaching a cell-like concentration and compared the protein behavior observed with that induced by PEG500, a synthetic crowding agent. Despite some similarities between the environments, protein crowders showed a number of characteristics that raise concerns about the use of diluted solutions or synthetic agents when studying protein behavior.

## Introduction

Most *in vitro* and *in silico* biophysical experiments treat proteins as highly purified entities that act in isolation, overlooking their natural “habitat”, namely the cell cytoplasm. This “habitat” contains between 80 to 400 g/L of several other macromolecules, which together account for 5%-30% of volume occupancy [1]. Among the effects that a crowded environment exerts on protein behavior, volume exclusion is considered the most relevant [2]. Accordingly, crowders behave as inert molecules that do not interact with proteins, and their presence limits accessible space to proteins, thereby reducing the conformational entropy and favoring compact folded forms of the latter [3]. Following this view, most experimental studies on proteins in dense environments have been performed by adding large polymers, such as poly(ethylene glycol) (PEG), Dextran or Ficoll, to the media. These polymers, often referred to as “inert” crowders, are assumed to exclusively mimic the volume-exclusion effect [4]. However, recent experiments show that “inert crowders” exert a complex variety of effects on protein stability, and results largely dependent on the type and size of the crowder involved [3,5,6]. For example, calorimetric analysis concludes that Dextran, glucose and PEG lead to an enthalpic stabilization and an entropic destabilization of the protein; the latter predominant only in presence of PEG [7]. Indeed this synthetic compound appears to be less “inert” than expected due to attractive interactions with proteins, questioning its effectiveness in recreating a pure volume-exclusion effect. Despite so, PEG continues to be used as a reference agent to model macromolecular crowding [8,9]. Regarding the size, intuitively, the volume excluded by inert crowding agents is proportional to the crowder size and consequently small crowders might even help unfolding [5]. Recent studies in cell-like environments have further challenged such a model, suggesting that compacted conformations of proteins may not always be favored in physiological crowded environments [9–15].

Available data suggest that protein crowders have a dual nature. On the one hand, they display the classical volume-exclusion effect and, on the other, they have the ability to form weak and transient (quinary) soft interactions with solute protein [9,11,12]. These effects generates competition between destabilizing and stabilizing forces, the final result of which is difficult to predict [11,13,14]. To further complicate the scenario, crowding might also affect the folding landscape, leading to alternative states not present in dilute solutions and affecting protein functionality [15]. This distortion of the conformational landscape might have a dramatic impact on highly dynamic proteins, such as intrinsically disordered (IDPs) and molten globule proteins (MGPs) [16]. Unfortunately, most crowding studies performed with these proteins have used synthetic polymers and often report only the expected increase in the compactness of the structure [17–21]. Research into IDPs or MGPs in cell-like crowded environments is more rare and provides unclear conclusions [10], [18], [28–33].

A consensus theory—based on experimental data—on the nature of crowding is impeded by the intrinsic limitations of studying highly dynamic systems in which single molecule information is lost within the experimentally detected structural ensemble [20–26]. Theoretical calculations, particularly molecular dynamics (MD), give direct access to atomic information on single molecules in carefully controlled environments, and they are therefore the perfect complement to experimental ensemble-based techniques when addressing crowding effects [21], [35–38]. Here we took advantage of the power of MD simulations to explore the impact of small-sized synthetic (PEG500) and protein crowders (proteins) on the structure, dynamics and interactions of the following three proteins: i) an intrinsically ordered protein (IOP), the 191-residue interferon regulatory transcription factor (IRF-3); ii) a molten-globule conformation (MGP), the 51-residue nuclear coactivator-binding domain of CREB (NCBD); and iii) an intrinsically disordered protein (IDP), the 47-residue activator for thyroid hormone and retinoid receptor (ACTR). These three proteins not only model the three major types of protein conformational landscapes, but also define a specific biological network, with NCBD as the central partner (the hub), able to transiently interact with IRF-3 and ACTR, thanks to its structural promiscuity [39–42]. This is the first study to present calculations of the effect of crowding on proteins of distinct structural complexity that define a biologically relevant crowded microenvironment.

## Results

**Fig 1:**
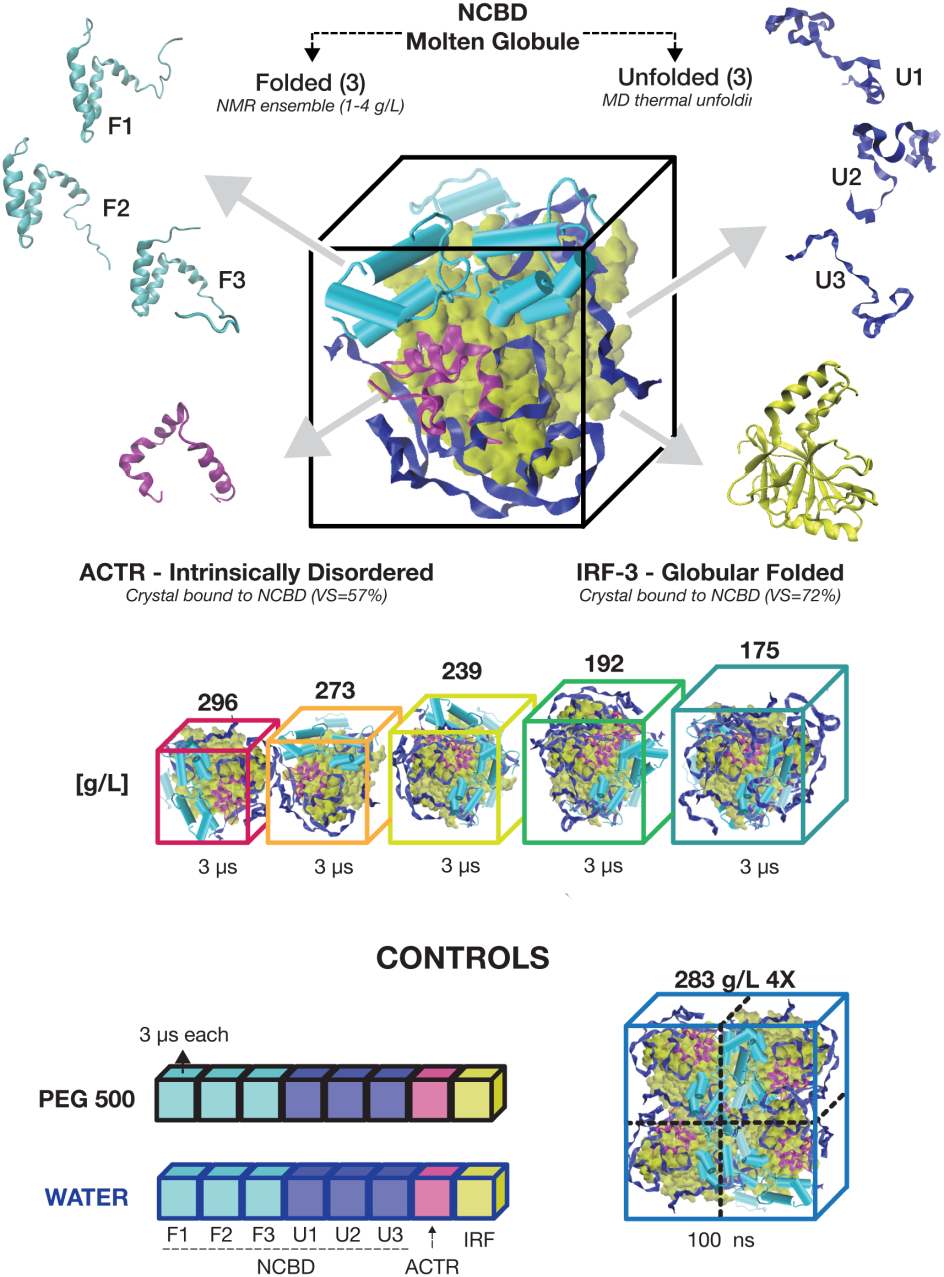
The simulated crowded systems. From the top: example of one of the simulated boxes (192 g/L) composed by eight structures: three conformations of NCBD from the folded NMR ensemble (PDB 2KKJ); three unfolded conformations of NCBD from a simulation at 500K, one conformation of ACTR, (PDB: 1KBH), and one conformation of IRF-3 (PDB: 1ZOQ); the five concentrations used as protein crowders; and the control simulations. Below each box, the minimum simulated time is indicated. More detailed listing of simulations performed can be shown in S1 Table.

We performed microseconds-long MD simulations of five crowded systems, each composed by eight conformations of the three protein types (6 NCBD, 1 ACTR and 1 IRF-3) at increasing concentrations [from 175 to 300 g/L] (Fig 1). Each conformation was individually simulated in solution with the synthetic crowder PEG500 and in water. The latter condition was used as a control of the behavior of proteins within the selected simulation protocol.

### Control simulations in water

**Fig 2:**
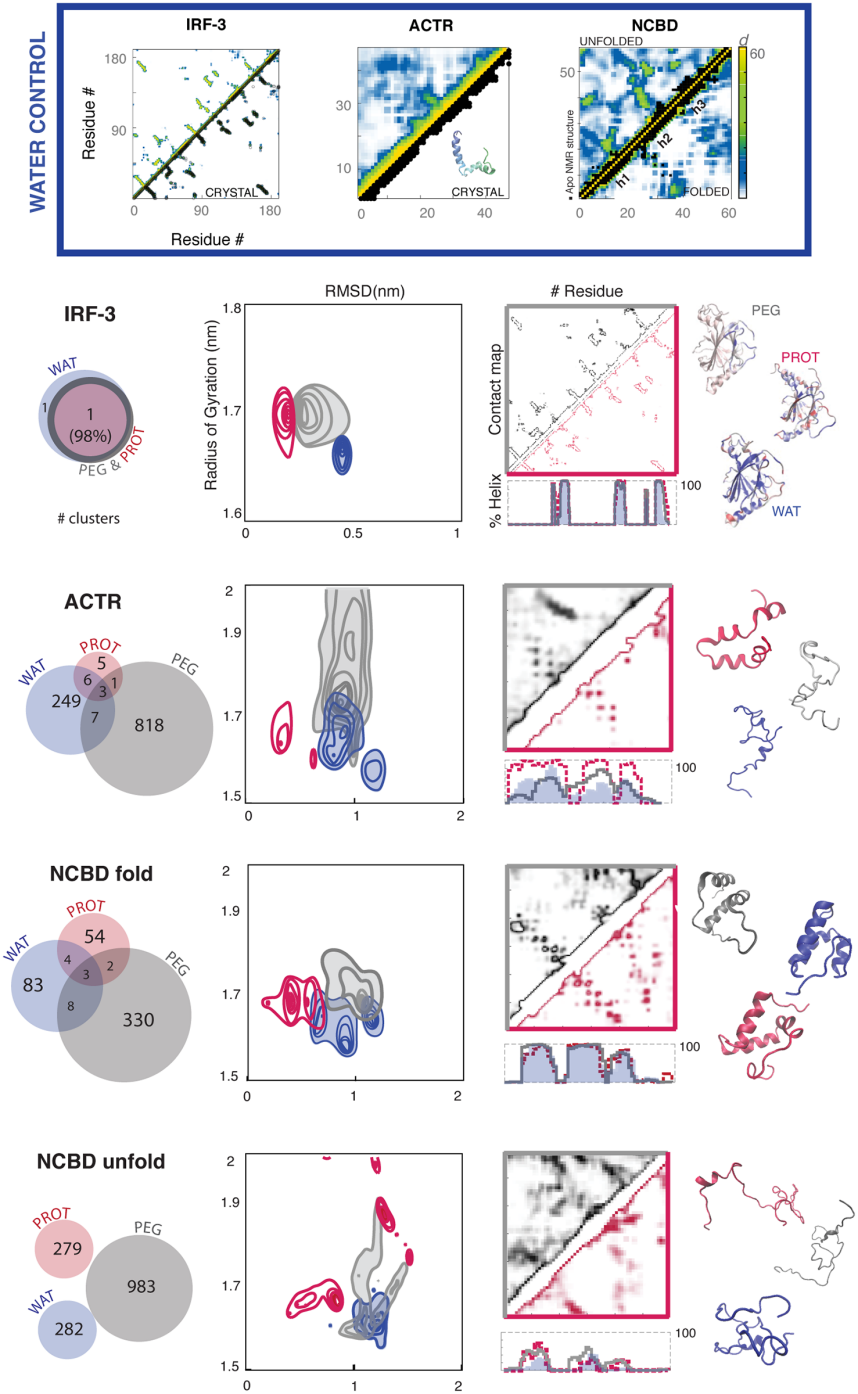
Structural changes in the three simulated environments. Top panel: contact maps in water, against reference PDB structure (in black) for the IRF-3 and ACTR; in the case of NCBD, contacts in the folded vs. unfolded trajectories are shown. Bottom panel from left to right: conformational overlap between the clusters of each simulated environment; sampling maps based on the RMSD values from the starting conformation (x-axis) and the radius of gyration (y-axes); contact maps for the two crowded environments (see S1 Fig for water) and helical content along the sequence (calculated with STRIDE); and structures representing the most populated cluster in each environment. Color code: blue for water, red for crowding at a concentration of 192 g/L, and grey for PEG500 (200 g/L). For NCBD, the values from all the three conformations (three folded and three unfolded) are grouped together.

Trajectories in pure water (S1 Fig and Fig 2) showed the expected behavior for the proteins under study. Thus, the intrinsically ordered protein (IOP: IRF3) was stable during the entire trajectory, maintaining the pattern of secondary structure, fold and shape. Native contacts were well preserved, with sizeable movements localized only at the C-terminal helix, in a region with interface contacts in the crystal. A small, but detectable, tightening of the hydrophobic core of the protein occurred. The intrinsically disordered protein (IDP: ACTR) was extremely mobile in water, sampling a wide repertoire of conformations. In this regard, clustering analysis detected more than 250 distinct conformers (most of them compact; see S1 Fig), none of which populated more than 5.5% of the trajectory. The contact map was fuzzy (compare with IRF3 in S1 Fig and Fig 2), suggesting the absence of remote long-lasting contacts, thus hindering the formation of stable folds. Some segments of ACTR tended to form a secondary structure, especially an α-helix at the N-terminal—an observation that is consistent with the results from NMR experiments [32,33]. However, these helical elements were unstable and fuzzy, with local populations rarely above 50% and undefined boundaries, making them unable to nucleate the global structure. Finally, the molten globule protein (MGP: NCBD) showed slow diffusion along the conformational space, with strong memory effects in the trajectories [30,34–36]. When the NCBD trajectory started from the “folded” conformation, significant plasticity was observed (around 100 structural clusters). This plasticity is attributed to the distinct orientation of the three helical motifs (h1, h2 and h3, see below and S3 Fig), which generate a fuzzy contact map with helical arrangements of the prevailing ACTR-binding form, while the helical arrangements required for IRF-3 recognition were rare. When the NCBD starting conformation was “unfolded”, it rapidly collapsed into an amorphous globule; the protein formed many remote and unstable contacts (282 structural clusters), and only small nascent elements of secondary structure (particularly in h1 and h2) were observed. NCBD appears to be a protein that was not evolutionarily designed to collapse into a single well-defined minimum. We conclude that control simulations provide a reasonable picture of the conformational landscape of the three proteins representing IOPs, IDPs and MGPs in water. We can therefore confidently use the same force-field and simulation protocol to explore crowded environments.

### Synthetic vs. protein crowders

As described above, most theoretical and experimental studies on crowding have been performed using polymers (as PEG500) as co-solvents, which theoretically act as “inert” crowders mimicking cellular crowding. However, whether polymers such as PEG500 are truly “inert” crowders and whether they correctly mimic the crowded environment in the cell remain to be confirmed. In order to answer these two questions, we compared the trajectories of the three model proteins in water, and in PEG500-crowding and protein-crowding conditions (using similar crowder concentrations in both cases) (Fig 2).

For IOP (IRF3), the effect of crowding was modest, and neither proteins nor PEG500 induced large changes in the local or global structure of this well-structured protein. Crowding stabilized the secondary structure, including the C-terminal helix, which was fragile in the simulations in water. When compared to water, both types of crowders produced an increase in the size of the protein (see Fig 2 for radius of gyration, and S2 Fig for solvent-accessible surface). This observation is not consistent with the “exclude volume” theory. Only protein crowders were observed to decrease the relative ratio of polar solvent-accessible surface, thereby suggesting that they attenuate the hydrophobic effect compared to water, the latter environment showing a more visible collapse of the core (cartoons in Fig 2 and S2 Fig). Interestingly, the crystal structure of IRF-3 was more similar to the conformations sampled in a crowded environment (especially in the protein media) than to those in dilute aqueous conditions. These findings thus suggest that crystals can, in some cases, mimic physiological conditions better than water.

For IDP (ACTR), crowding agents had a huge impact on the conformational landscape, (Fig 2); however, we were unable to find a pattern of general “crowding” effects, since the changes induced by PEG500 differed from those induced by a protein environment. Thus, PEG500 generated a large expansion of the sampled conformational space, which became dominated by extended conformers showing only a moderate amount of secondary structure. In contrast, protein crowders reduced the conformational space sampled, which was now dominated by relatively compact conformations, with well-defined α-helices localized in those regions required for NCBD binding [37,38]. These results demonstrate the inability of PEG500 to reproduce physiological-like crowded conditions around IDPs and suggest that protein crowding might contribute to IDP folding in the bioactive conformation.

For MGP (NCBD), the behavior of crowders largely depended on the starting conformation, mirroring the “memory effects” detected in the simulations in water and reinforcing the idea that NCBD (and probably other MGPs) moves across a complex and frustrated conformational landscape. In the trajectories starting from folded NCBD, crowders favored more extended conformations than those sampled in water, introducing significant changes in the fuzzy pattern of long-range contacts (Fig 2). The helical fragments were often arranged in the bioactive conformations, sometimes closer to the IRF-3-bound state, which has never been sampled in water (S3 Fig). The bias towards the bioactive state was especially visible for protein crowding, where collected ensembles were on average 0.34 nm closer to the bioactive conformation found in the NCBD-IRF-3 complex than those sampled in water. The effect of crowders was even more dramatic (and complex) for NCBD trajectories starting from an unfolded state. Both PEG500 and protein crowders hindered the hydrophobic collapse observed in water, thus favoring extended conformations (Fig 2) in which native helices—which were hardly distinguishable in water—showed significant populations and well-defined boundaries (especially for helix 1). These observations again support the notion that crowding might help disordered proteins to adopt bioactive conformations. When analyzed in detail, the effects of synthetic (PEG500) and protein (protein) crowding differed significantly (Fig 2), thus again raising concerns about the use of small-sized PEG as a model of physiological crowding.

Overall, compared to water, both synthetic and protein crowders favored open and moderately extended conformations with higher secondary structure content. These results are difficult to explain on the basis of the “excluded volume” hypothesis. The breakdown of the energies between each protein and its surroundings reveals that in presence of both protein and PEG500 the Van der Waals term (Lennard-Jones) increase its weight compared to dilute solutions, at the expense of Coulomb interactions (see S2 Table). However, the percentage of the vdW term in PEG500 is smaller but comparable to the one in protein crowding (~ 21.5% and ~ 19.5% respectively in protein crowding and in PEG500 for NCBD, ~ 16% and ~ 12.5% for ACTR, and ~ 13% and ~ 12.5% for IRF-3), confirming that PEG500 is a non-inert crowder. In general crowding behaves as an unexpected partner, favoring protein binding through the conformational selection paradigm and acting as a chaperon that modulates the conformational space of non-ordered proteins.

### Concentration effects on crowding

**Fig 3:**
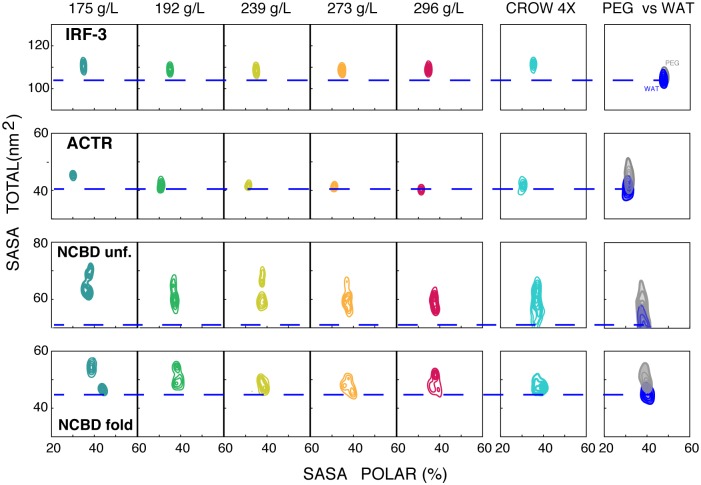
Changes in the solvent-accessible surface area (SASA) of the protein in crowded systems. a) Sampling maps of the percentage of polar SASA (x-axis) and its total (y-axis) in nm^2^ calculated for the five concentrations of crowded systems and the other controls (CROW 4X = 100 ns at 182 g/L of a 4 times larger system, PEG500, water). For NCBD, the values of all three conformations (three folded and three unfolded) are grouped.

The analysis of 5 independent trajectories obtained at protein concentrations from 175 to 296 g/L showed that the conformational landscape of the proteins was relatively robust to moderate changes in the concentration of the protein environment. However, detailed analysis revealed some subtle, but systematic, concentration-dependent changes (see Fig 3, and S4–S6 Figs). For example, a low concentration of protein crowders favored extended conformations, while increasing concentrations favored more collapsed structures (Fig 3). This observation suggests that the effect of protein crowding results from the combination of two opposing contributions: i) soft protein-protein interactions, which favor the exposure of protein moieties and the prevalence of extended conformations; and ii) the “excluded volume” effect, which favors collapsed structures. At low and moderate protein concentrations, the first effect dominates; however, as the number of possible protein-protein contacts is satisfied, the “excluded volume” effect gains relevance, leading to more collapsed structures. The navigation of proteins above their energy landscape can then be fine-tuned by modifying the protein concentration in different cell compartments, thereby creating an additional layer of regulation of protein structure and function.

### Protein quinary contacts and crowding

**Fig 4:**
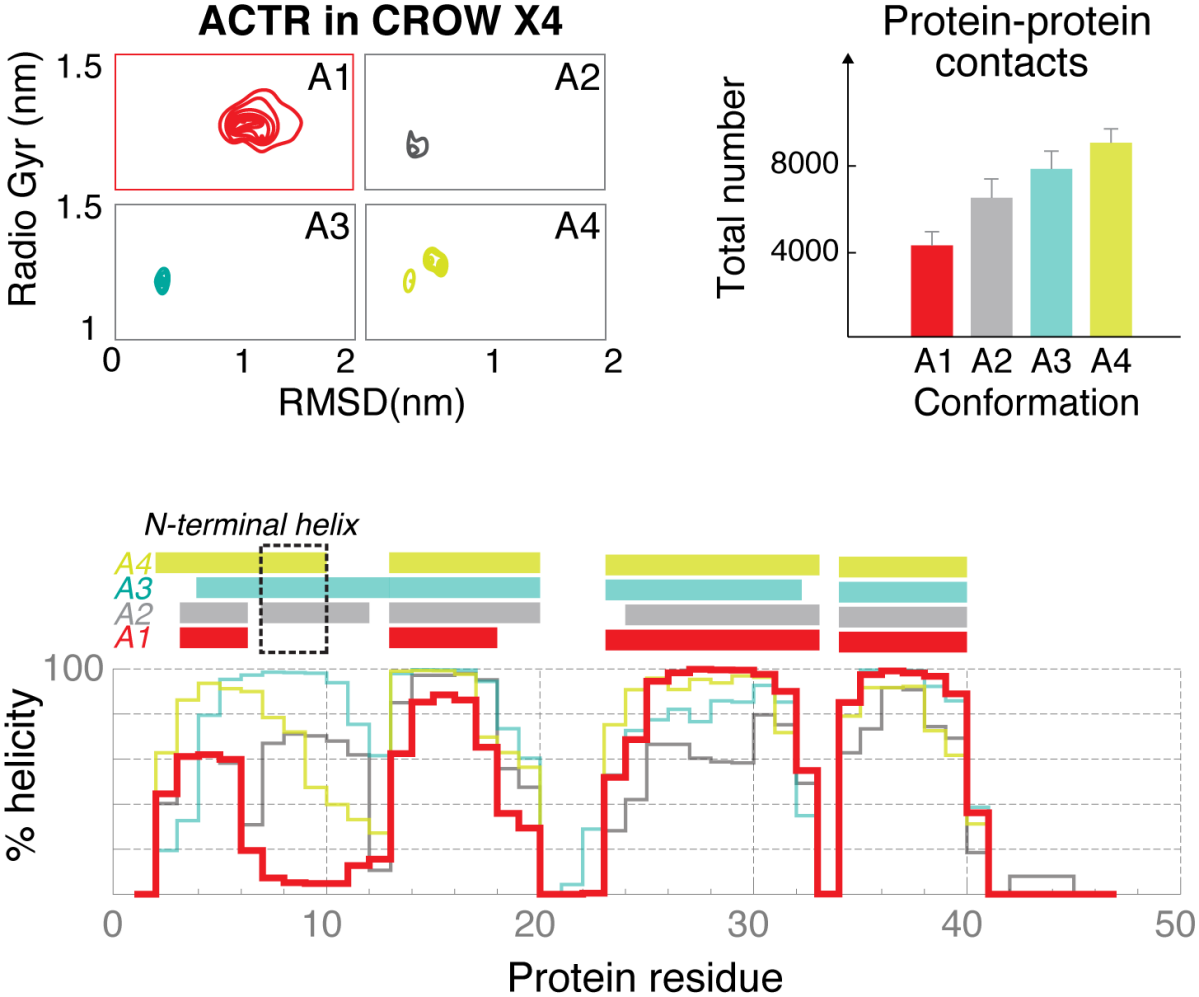
Structural descriptors for the four conformations of ACTR in the 4X box. a) Frequency maps of the RMSD values from the starting conformation (x-axis) and the radius of gyration (y-axis) in nm. b) The total number of inter-protein contacts is reported. c) Helical content (%) along the sequence.

The results above strongly suggest that soft protein-protein interactions are responsible for the crowding effect generated by a dense protein environment. A key question is whether these contacts correspond to unspecific transient (quinary) or specific interactions, the latter could not be *bona fide* annotated as crowding. To study this point, we compared the 3 replicas of NCBD (both for the folded and unfolded ones), where NCBD has different protein neighbors. If specific protein-protein interactions play a major role in modulating protein behavior, we can expect the 3 replicas to show distinct behaviors. This was not found to be the case (S3 Table, S4 and S7 Figs); specific interactions can therefore be ruled out as a major guide of the simulations. To further confirm this point, we performed additional trajectories with a 4x larger simulation box (4X CROW; 182 g/L protein concentration), which provided us with several replicas of the different proteins. Again, no remarkable differences were found between the sampling obtained here and the one in smaller simulation boxes (Fig 3, S5 and S8 Figs and Fig 4). Interestingly, the only remarkable exception was one of the copies of ACTR with an N-terminal exposed to a region of low protein density (labeled as A1, in red in Fig 4). There, the lack of protein-protein contacts caused an immediate response (within 100 ns) in ACTR, which underwent structural rearrangements (loss of helicity in the N-tal). These were not achieved when ACTR was surrounded by proteins. In summary, unspecific rather than specific protein-protein contacts appear as a major determinant of the effect of protein crowding.

**Fig 5:**
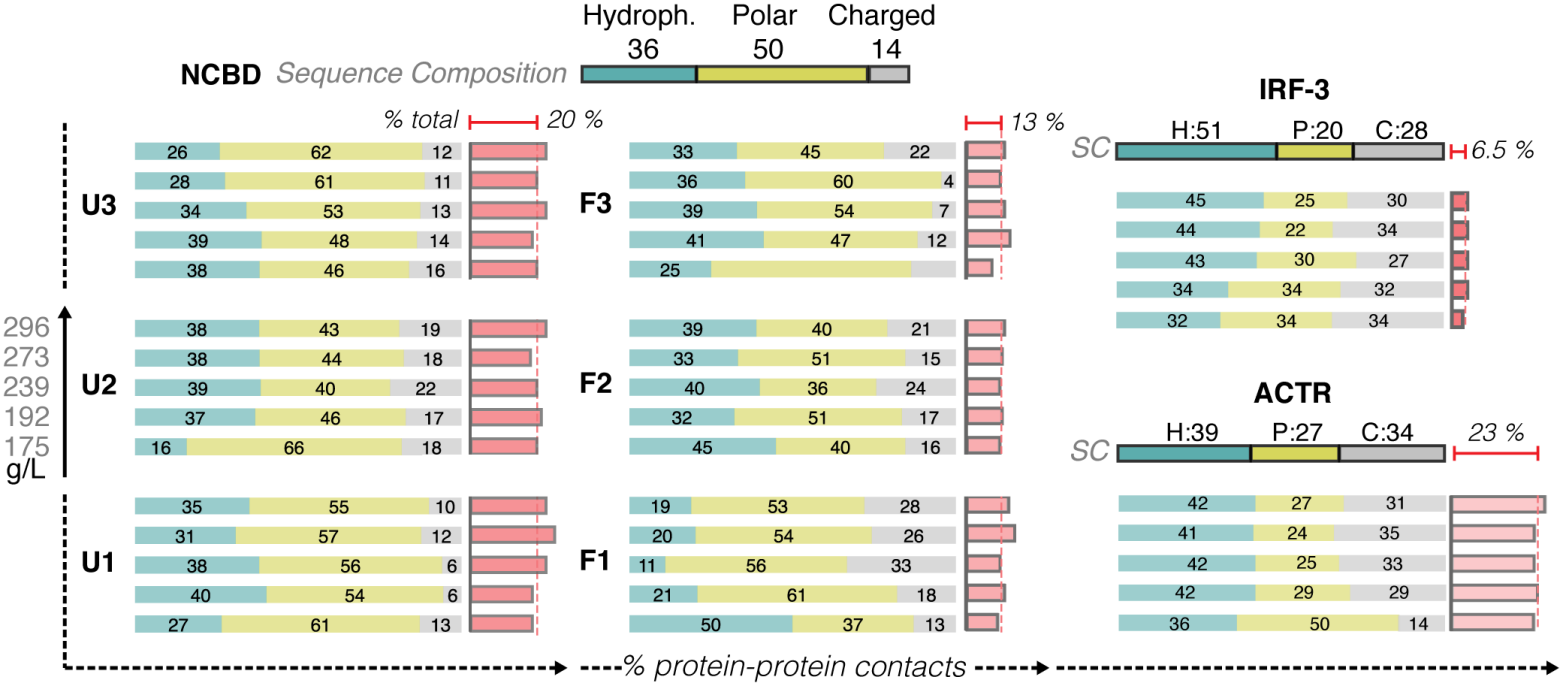
The non-specific protein quinary contacts in crowded environments. For each conformation, the distribution of the inter-protein contact on the basis of the nature of the residues involved is reported at increasing protein crowder concentration (down—top). The darker boxes on top show the reference values of the protein sequence (H: hydrophobic in blue, P: polar in yellow and C: charged in gray). The percentage of the inter-protein contact of the total (inter and intra) is also reported in red; the average for each protein is reported at the top (see also Fig 6).

**Fig 6:**
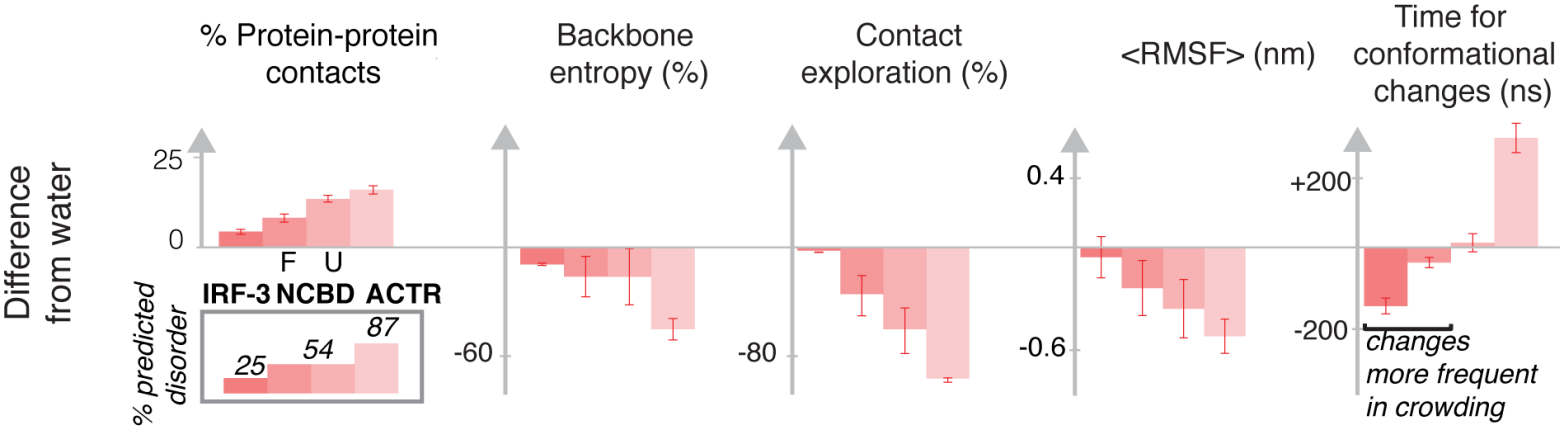
Effects of protein crowding depend on the protein disorder. From the left: the percentage of protein-protein contacts (from the total contacts that a protein forms) is proportional to the intrinsic disorder of each protein (calculated with PONDR-FIT) [39]; the same trend is followed by the other observables that report the decrease in the conformational exploration compared to the simulation in water (calculated globally as the backbone conformational entropy and locally as the % of explored intra-protein contacts;) and the change in protein dynamics (calculated locally as the average local root mean square fluctuation RMSF (nm) and globally as the time between conformational changes). Values are averaged from the crowder concentrations. This trend was not observed in PEG500, see S11 Fig for a comparison.

The crowding shown here had a higher presence of disordered proteins, which were generally characterized by a higher content of charged residues. However, we did not find any significant enrichment in the type of residues located at the contact regions or any dramatic concentration-dependent changes in the inter-protein contacts (Fig 5). Intriguingly, the number of protein-protein interactions and the preference for protein vs. water contacts rose as the intrinsic disorder of the protein increased (Fig 6 and S3 Table). This observation explains why crowding effects are especially dramatic in disordered proteins. In summary, we conclude that our simulations reproduce *bona-fide* “crowding effects”, which are not contaminated by specific interactions that might occur in a biologically relevant cluster (IRF-3, ACTR and NCBD).

### Promiscuity and frustration

As described above, the presence of a protein environment helps the protein adopt conformations that more closely resemble bioactive ones; however, it also generates contact frustration, as the prevalence of non-specific quinary contacts hinders specific partner recognition. This frustration becomes evident by analyzing the interactions between NCBD (a total of 40 trajectories of NCBD were collected) and its partners (ACTR and IRF-3). All the crowded systems failed to reproduce the contacts observed in the experimentally solved complexes (S9 Fig). When binary complexes (NCBD:ACTR and NCBD:IRF-3) were placed in water, they rapidly adjusted to form new contacts. Remarkably, each complex rebuilt a similar pattern of contacts in most of the simulated copies in water (8 out of 10 for ACTR / F1 and 7 for ACTR / U2, see S10 Fig), thereby suggesting that these intrinsically favored contacts are frustrated in crowded conditions as a result of the presence of many competing interaction partners. Overall, the crowded box appeared as a stagnant system, where contact promiscuity generated a frustrated pattern of interaction that hindered the formation of bioactive conformations.

### The impact of crowding on dynamics

Protein crowding limited the accessible configurational space both globally (as noted in the number of recognized clusters) and locally (as noted by the fuzziness of the intra-protein contacts) (Fig 2 and S2 Table). The greater the “in-water” intrinsic disorder of a protein, the larger the effect of protein crowding in slowing down protein dynamics (see Fig 6 and for PEG500, where this effect was not observed, S11 Fig), Despite so, the protein underwent frequent but small oscillations that did not produce major conformational changes. As expected, the presence of protein crowders led to a significant increase in viscosity, which was reflected in the reduction of atomic movements. For example, the diffusion of water molecules was slowed down by ~ 25% from a pure aqueous environment (Table 1) [27], while global protein diffusion was reduced to 1/10 of the original value and within the same range as that reported in other studies (below 10X) [40–43]. Note that diffusion values in crowded environments should not be considered as quantitative predictions [35],[44], [51]; however, given that our results confirm those of other studies, we are confident that they still provide a valid qualitative insight. Indeed the impact on diffusion rates and related binding kinetics [8,43] is known to affect the basic functionality of the protein [40].

**Table 1:** Diffusion coefficient of water molecules and proteins in a crowded environment and in water. The table reports the average diffusion coefficients and their standard deviations for all the water molecules and all the proteins present in the simulated box.

	Diffusion Coefficient [μm^2^ / s]		
	Water		Proteins
**175 g/L**	4900	(120)	11
**192 g/L**	4847	(199)	10
**239 g/L**	4291	(91)	7
**273 g/L**	4219	(162)	8
**296 g/L**	4041	(123)	11
**WATER**	5108	(226)	86

## Discussion

Our study provides a comprehensive picture of the impact of crowding on the conformational space of three proteins with different structural levels: well-structured, intrinsically disordered, and molten globule. Compared to dilute solution, both small-sized synthetic (PEG500) and protein crowders favored open and moderately extended conformations with higher secondary structure content. However, proteins in PEG500 experience a larger increase in conformational entropy, confirming observation from calorimetric analysis in presence of PEG molecules with similar dimension [7]. Overall we join the concerns regarding its employment in macromolecular crowding: detailed analysis showed that, in general, there were few similarities between the effect of PEG500 and protein crowders. This finding thus argues against the generalized use of PEG of low molecular weight to simulate crowding in physiological environments.

Our results suggest that as previously suggested in previous studies [7,11–14] the protein crowding represented here is a battlefield between two opposing forces, namely the soft protein-protein interactions and the “excluded volume” effects; the outcome depending on the concentration and type of crowder involved. Interestingly, the impact of such protein crowding strongly depends on the intrinsic structural level of proteins (in water). We found that crowding leaves the overall structure of folded proteins (such as IRF-3) almost unaffected but reduces their collapse into the hydrophobic core. This observation would explain the small expansion of the protein and the agreement with the available structures from crystals compared to pure water simulations. The impact of crowding on non-structured proteins was found to be more dramatic and complex, and it typically led to a gain in structure, bringing it closer to the ensemble of bioactive conformations and therefore favoring the conformational selection paradigm of protein binding.

Protein crowders (but not PEG500) limit the exploration of new intra- and inter-protein contacts, leading to a global decrease in conformational entropy, balanced by the enthalpic stabilization cause by quinary contacts, in good agreement again with some of the existing models of crowding [7,11–14]. The prevalence of these soft, transient and non-specific protein-protein contacts slows down solvent diffusion, as well as the global and local dynamics of proteins, thus producing frustration in native contacts, which may slow down functional flexible proteins. Crowding favors bioactive conformations, which may facilitate conformational selection processes, but on the other hand hinders the formation of functional contacts, showing then a dual effect whose impact in functionality is difficult to predict. Although we were unable to detect any bias in the type of contacts formed in the crowded box, we cannot exclude bias in the results caused by the high presence of highly charged disordered proteins. Further studies might address the differential impact as crowders for disordered and ordered proteins and discard eventual IDP-driven artifacts, as these proteins have a unique sequence composition that keep them unfolded under physiological conditions, which might bias their crowding properties. Additional work is also required to evaluate the ability of longer “inert” polymers to simulate protein crowding, as PEG500, which is very convenient to perform MD simulations shows a molecular volume much smaller than that of average proteins. We could expect that longer polymers might simulate better the crowding properties of proteins.

The cell interior differs from an aqueous dilute environment. However, it is far from a “bag full of molecules”and is possibly organized into compartments in which proteins are stabilized or destabilized in response to the specific surrounding environment [45], thereby creating an unexpected extra level of regulation of protein functionality, especially in the case of the ultra-sensitive IDPs, whose structure and dynamics can differ depending on the cellular context. From this perspective, crowding can be regarded as a collective chaperon that modulates protein conformational space.

## Methods

### Overview of the crowded models

We mixed NCBD, ACTR, and IRF-3 to obtain five dense (175, 192, 239, 273 and 296 g (of protein)/mL: protein volume fraction 20–30%) protein solutions. A stoichiometry of 6:1:1 (NCBD, ACTR and IRF-3) was used to better reproduce the central protein of the system: NCBD, for which we considered 6 starting conformations (one per copy), three of them taken from a NMR ensemble (PDB: 2KKJ) and corresponding to “folded” states (F1-F3 in the remaining), while the other three were taken from a 50-ns MD simulation at T = 500K, corresponding to a fully “unfolded” protein (U1-U3 in the remaining). The starting conformations for ACTR and IRF-3 were taken from the Protein Data Bank (PDB entry IDs 1KBH and 1ZOQ respectively) without the bounded partner. To remove any bias from the simulations, the starting positions and orientations of the proteins in the simulation boxes were random (see below) and the distance between conformations was increasingly reduced to reach more dense environments. Water molecules were added to fill the box size, calculated with a decreasing distance from the proteins (from 0.5 to 0.1 nm) and the final density was then calculated considering the proportion between water molecules and proteins. See Fig 1 for a map of the simulations performed. All these simulations were extended for at least 3 μs of unbiased dynamics.

### Control simulations

Control simulations at a comparable timescale were performed in two environments: eight simulations (1 for ACTR, 6 for NCBD, and 1 for IRF-3) in pure water boxes; and eight additional simulations in a water:PEG500 mixture (200 g/L PEG500 concentration). In order to check for potential biases produced by the finite size of the simulation box and the use of a given set of relative orientations of the proteins, we performed one additional simulation with a ~4-times larger box containing 24 NCBD, 4 IRF-3 and 4 ACTR proteins. This huge system (~277,000 atoms at 182 g/L of concentration) was simulated for 100 ns, allowing us to collect information on each protein copy in many different surroundings.

To address the interaction of NCBD and its partners in a crowded environment, we extracted protein pairs formed by either a folded or an unfolded conformation of NCBD (F1 and U2 with ACTR, F3 and U3 with IRF-3) from the crowding simulation at 273 g/L and used them as starting seeds for multiple simulations in pure water and crowded conditions (273 g/L). For each of the four systems, 10 simulations of 10 ns were performed (reaching a total of 400 ns in water and in protein crowding respectively). These short times allowed us to exclusively study the fast relaxation of the potentially frustrated protein-protein contacts.

### Simulation set-up

All starting structures were titrated, neutralized with monovalent ions, hydrated, minimized, thermalized, and pre-equilibrated using our standard procedure implemented in the MD-Web server [46]. In the case of PEG500 systems, proteins were immersed in a pre-equilibrated box of water/PEG molecules at a concentration of 200 g/L (starting PEG500 conformation PDB ID 4APO); the resulting systems were then pre-equilibrated by relaxing solvent for 10 ns prior to the general MD-Web equilibration procedure [46]. Unless otherwise stated, all the trajectories were collected with Gromacs 4.5 [47] using a time step of 2 fs in the isothermal (300 K) and isobaric (1atm) ensemble with Nose–Hoover thermostat and Berendsen barostat [48–50]. We applied periodic boundary conditions and particle Mesh Ewald corrections [51] for the representation of long-range electrostatic effects with a grid spacing of 1.0 nm and a cut-off of 1.0 nm for Lennard-Jones interactions. Constraints on chemical bonds were solved by the SHAKE algorithm [52]. The Parm99-SB-ILDN force field was used for proteins [53], TIP3P for water molecules [54], and modified TraPPE-UA parameters described by Fischer and colleagues for PEG molecules [55].

### Analysis

Gromacs standard routines and analysis tools in MD-Web [46] were used to analyze the trajectories, with a minimum resolution of 20 ps. We evaluated overall protein compactness using the radius of gyration (Rgyr), the deviation from a reference structure with the root mean square deviation (RMSD), the exposed surface to the outside with the solvent-accessible surface area (SASA), and the movements of each residues with the root means square fluctuations (RMSF). The secondary structure was evaluated by STRIDE [56], while VMD was used to visualize molecules and to analyze contacts [57]. The Coulomb and Lennard-Jones energy terms were calculated using GROMACS energy groups for each protein against the rest of the system. Inter- and intra-protein contacts were defined by a cutoff of 0.8 nm between alpha Carbons (Cα). Intra-protein contacts were defined as “explored” when they were found in more than five frames. Conformations recurrently sampled were detected by a two-step clustering of backbone atoms using the standard GROMOS algorithm [58]: first we reduced the total number of conformations in each trajectory with a cutoff of 0.15 nm, and then, for each protein, the reduced ensembles in WAT, PEG500 and CROW were collected together and subjected to a second clustering with a cutoff of 0.35 nm. Following Knott-Best [34], the relative orientation of the helices of NCBD was calculated by the relative elevation and azimuth between the helix vectors, defined with the axis formed by the Cα atoms in the helix of the PDB structure. The translational mean square displacements (MSD) of the center of mass of molecules were calculated to gain information on intermolecular movements (time windows of 10 and 25 ns were used for water and proteins respectively). Self-diffusion coefficients were determined using the Einstein relation, as described elsewhere, and periodic box corrections were applied [59]. Conformational entropies were approximated at the quasi-harmonic level using the last 1 μs of the simulations [60]. Finally, to detect conformational changes, we clustered the all-atom trajectory using the GROMOS algorithm [58] with a cutoff of 0.15 nm (0.1 nm for IRF-3), labeling any change in the cluster as a large conformational change.

## Supporting Information

S1 Fig The control simulations in water. For **a)** IRF-3 **b)** ACTR and **c)** the six conformations of NCBD are displayed: the RMSD evolution in time; the helical content along the sequence (blue boxes represent the helices found in the starting structure) and the cartoon-like representation of the most populated clusters (with the relative population reported below).
(TIF)

S2 Fig Changes in the solvent accessible surface area of IRF-3. The change is calculated as the difference in SASA values from the last to the fist frame for each protein residues. Residues with a positive difference, in red, are more expose to the solvent at the end of the simulated time, while residues with negative values, in blue, loose solvent exposure. Results are displayed for water, crowding at 192 g/L and PEG500. The table below displays the same difference in SASA, but classified into 5 groups according to the type of atoms involved: ALL (all atoms), SIDE (atoms in the sidechains), BB (atoms in the backbone), APOL (all non-oxygens and non-nitrogens atoms in the sidechains) and POL (all oxygens and nitrogens in the sidechain).
(JPG)

S3 Fig The position of the three helices of NCBD. **a)** A scheme to explain how elevation and azimuth are calculated from the helix vectors h1-3 as seen in [34]. **b)** A cartoon-structure of NCBD with the helix vector h1-3 marked as arrows. Notice the opposite positioning in two of the protein conformations. Each vector follows the principal axes of the atoms in the original helical region. The frequency of each specific helical conformation defined by Azimuth (x-axes) and Elevation (y-axes) is shown in **c)** for control systems and in **d)** for crowded system. Results are collected for the three folded conformations together. The black symbols define the values from the several NCBD structure available in the PDB: ACTR-bound (PDB: 1KBH), p53 bound (2L14), IRF-bound (PDB: 1ZOQ), the NMR ensemble of unbound NCBD (2KJJ) and the structure used as starting point.
(JPG)

S4 Fig RMSD in crowded environments. The evolution in time of the RMSD calculated from the starting conformation. Color code as in Fig 1: gray for PEG500; dark green to red for crowding concentration from 175 g/L to 296 g/L.
(TIF)

S5 Fig The sampling of RMSD and Radius of Gyration in crowding. The 2D sampling visualized with the RMSD values from the starting conformation (x-axis) and the Radius of Gyration (y-axis) in nm calculated in the five concentrations of the crowded system (from 175 g/L to 296 g/L.) and for the CROWDED 4x (182 g/L and four times bigger).
(TIF)

S6 Fig The effect of crowding concentration on two descriptors of protein structure. From the left: contact maps and the percentage of helixes along the protein sequence. In the case of NCBD, values are averaged for the three conformations (folded and unfolded). Color code as in Fig 1: gray for PEG500; dark green to red for crowding concentration from 175 g/L to 296 g/L.
(PNG)

S7 Fig Structural details for the six conformations of NCBD. For each conformation (U1-3 and F1-3) the contact map and the percentage of helixes along the sequence in a crowded environment (192 g/L—red) and in water (blue).
(TIFF)

S8 Fig Helical content in the 4X control. For each protein we compared the helical content calculated in all the conformations in the CROW 4X box (182 g/L) with the values taken from the crowding systems with comparable crowding concentration.
(TIF)

S9 Fig NCBD and its partners: Complex formation and contact frustration. Contact maps between NCBD residues (x-axes) and its two partners (y-axes—ACTR on the left and IRF-3 on the right). The plots in the first row display the contact time (% of the total simulated time) in the simulation at 273 g/L as an example of a crowded system. The black dots mark contacts in the bounded structure available at the PDB. The second row displays the difference (Water—Crowded) in contact time calculated in the 10 copies of 10 ns in crowded conditions and in water. The contact map calculated from the 10 copies at crowded conditions is plotted in the background to identify contacts gained from scratch in water. The latter are marked with grey boxes. The cartoons at the bottom illustrate contacts newly formed in water (left side) between ACTR (in magenta) and NCBD (in cyan) while the crowded environment (right-side) prevented their formation.
(JPG)

S10 Fig Contact maps of NCBD/ACTR complexes in water. For each complex (ACTR with F1 or U2) the contact maps for each of the 10 copies in water are shown. The red boxes highlight areas where new contacts (not present in the crowded environment) are formed in water.
(JPG)

S11 Fig The effects of PEG500 and protein disorder. Starting from left: for each protein the % of intrinsic disorder (calculated with PONDR-FIT) and several differences using the simulation in water as reference: the backbone conformational entropy; the % of explored intra-protein contacts; the average local RMSF (Å) and the average time between conformational changes (ns).
(PNG)

S1 Table Detailed listing of simulations performed in this paper. (ODT)

S2 Table Energies between the protein and the rest of the system. The table reports the percentage of Lennard-Jones energies on the total calculated as the sum of Lennard-Jones and Coulomb energies between each protein and the rest of the system.
(DOCX)

S3 Table Descriptors for the six conformations of NCBD. The table displays the average difference from simulation in water in several descriptors for each conformation of NCBD (Folded F1-3 and Unfolded U1-3) in presence of protein crowding (192 g/L) or PEG500. Values in bold are the average for each subgroup while the standard deviation is reported in brackets.
(DOCX)
