# Supplementary figures and images for: The Differential Response of Proteins to Macromolecular Crowding
Source: PLoS Comput Biol. 2016 Jul 29;12(7):e1005040. doi: 10.1371/journal.pcbi.1005040 (PMC4966950; doi:10.1371/journal.pcbi.1005040)

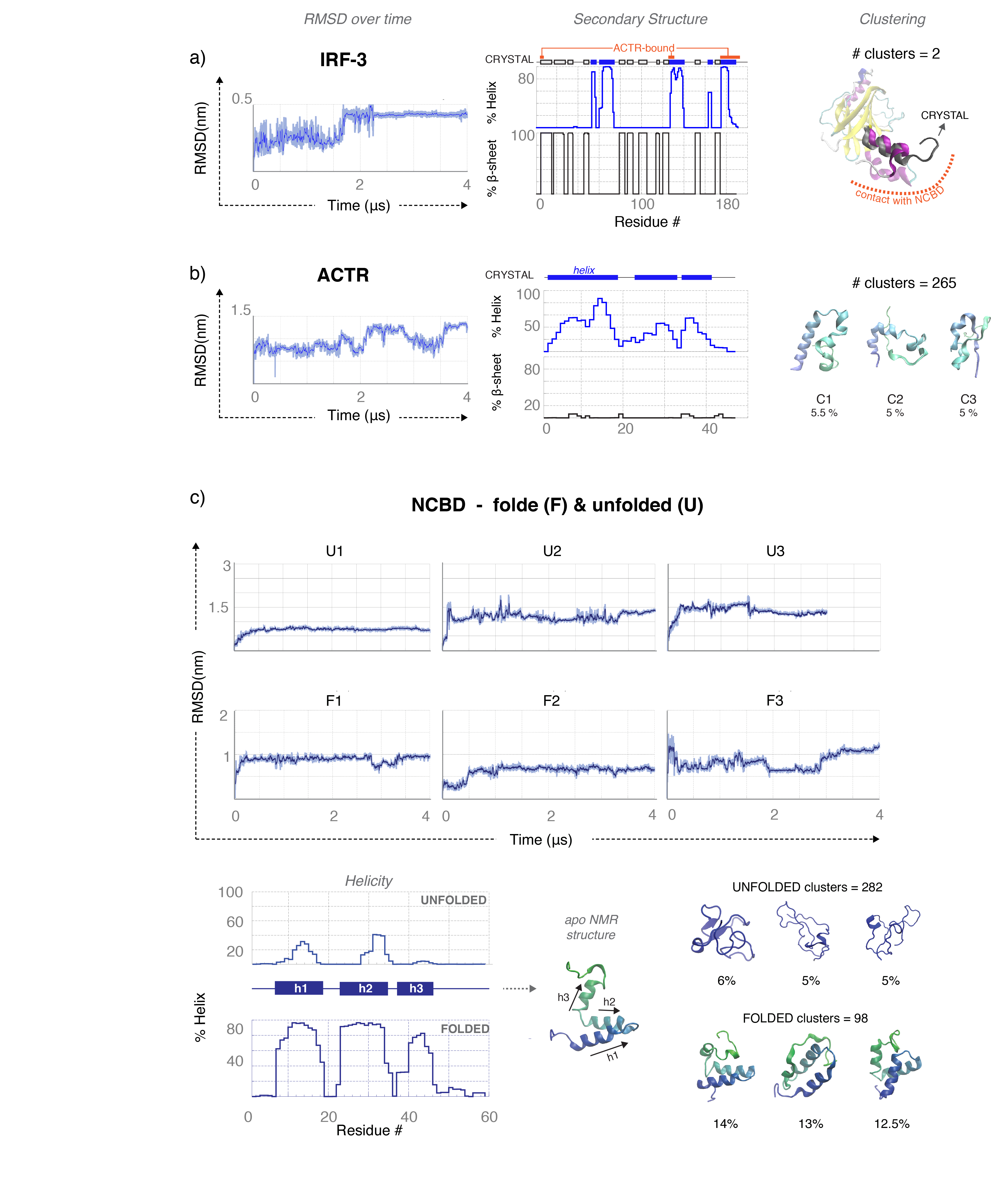

Supplement: S1 Fig — For a) IRF-3 b) ACTR and c) the six conformations of NCBD are displayed: the RMSD evolution in time; the helical content along the sequence (blue boxes represent the helices found in the starting structure) and the cartoon-like representation of the most populated clusters (with the relative population reported below). (TIF) [file pcbi.1005040.s001.tif]

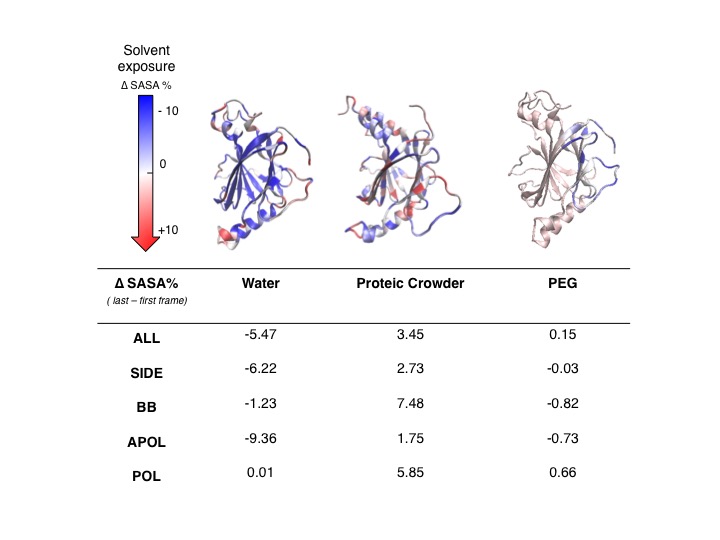

Supplement: S2 Fig — The change is calculated as the difference in SASA values from the last to the fist frame for each protein residues. Residues with a positive difference, in red, are more expose to the solvent at the end of the simulated time, while residues with negative values, in blue, loose solvent exposure. Results are displayed for water, crowding at 192 g/L and PEG500. The table below displays the same difference in SASA, but classified into 5 groups according to the type of atoms involved: ALL (all atoms), SIDE (atoms in the sidechains), BB (atoms in the backbone), APOL (all non-oxygens and non-nitrogens atoms in the sidechains) and POL (all oxygens and nitrogens in the sidechain). (JPG) [file pcbi.1005040.s002.jpg]

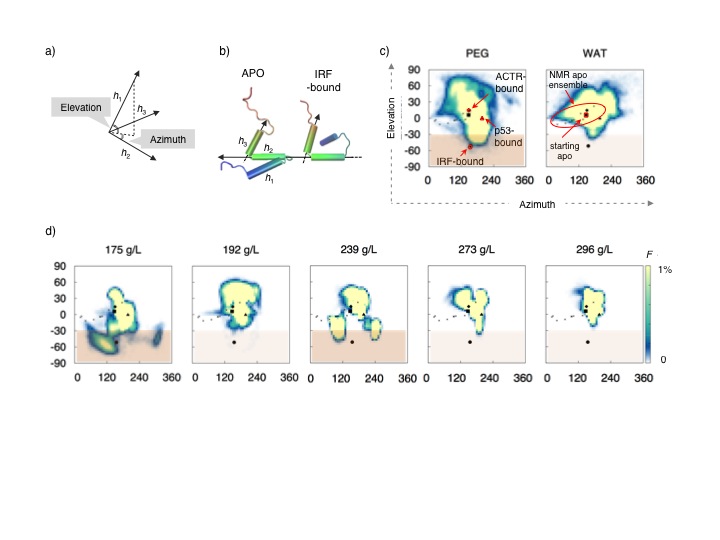

Supplement: S3 Fig — a) A scheme to explain how elevation and azimuth are calculated from the helix vectors h1-3 as seen in [34]. b) A cartoon-structure of NCBD with the helix vector h1-3 marked as arrows. Notice the opposite positioning in two of the protein conformations. Each vector follows the principal axes of the atoms in the original helical region. The frequency of each specific helical conformation defined by Azimuth (x-axes) and Elevation (y-axes) is shown in c) for control systems and in d) for crowded system. Results are collected for the three folded conformations together. The black symbols define the values from the several NCBD structure available in the PDB: ACTR-bound (PDB: 1KBH), p53 bound (2L14), IRF-bound (PDB: 1ZOQ), the NMR ensemble of unbound NCBD (2KJJ) and the structure used as starting point. (JPG) [file pcbi.1005040.s003.jpg]

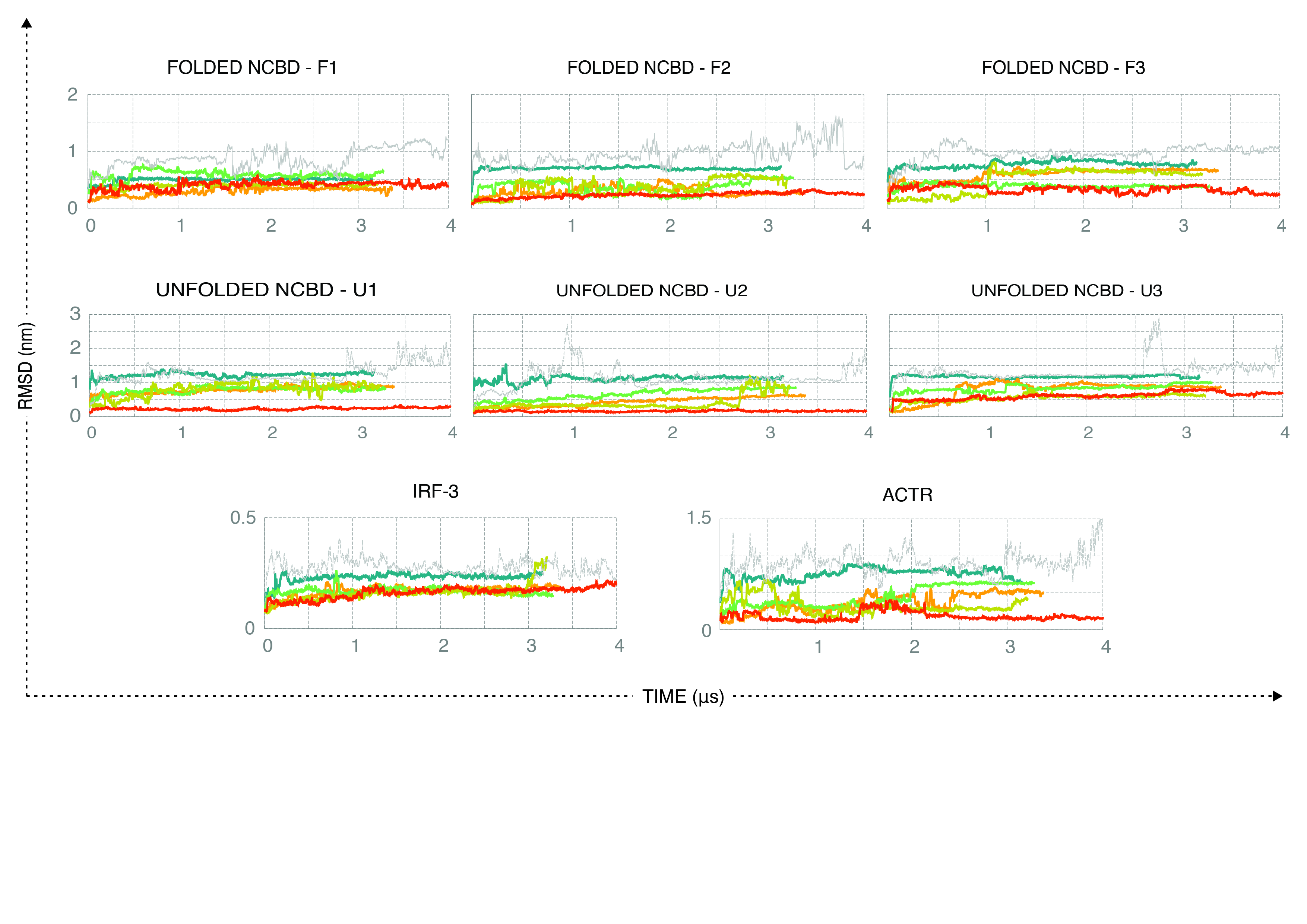

Supplement: S4 Fig — The evolution in time of the RMSD calculated from the starting conformation. Color code as in Fig 1: gray for PEG500; dark green to red for crowding concentration from 175 g/L to 296 g/L. (TIF) [file pcbi.1005040.s004.tif]

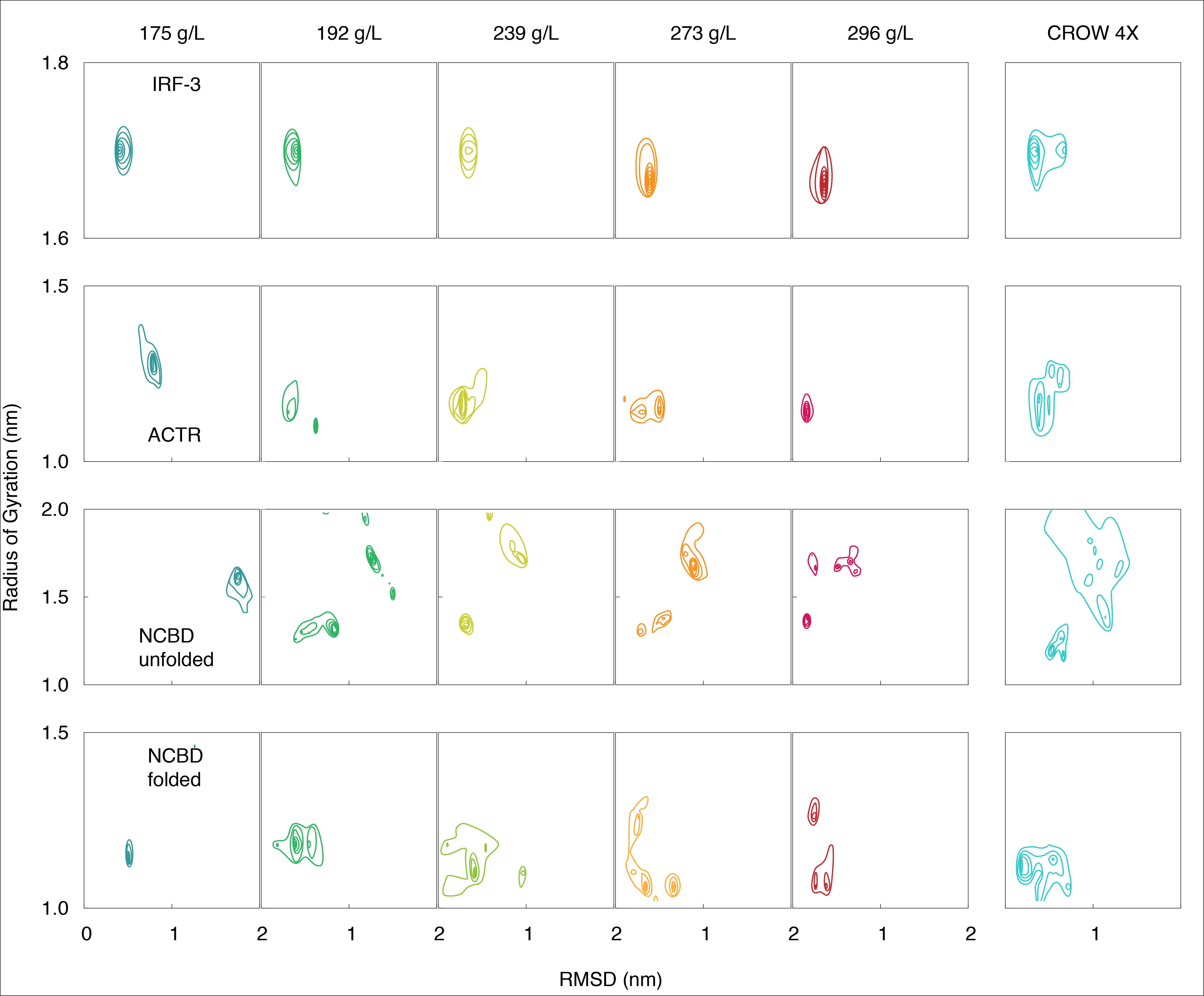

Supplement: S5 Fig — The 2D sampling visualized with the RMSD values from the starting conformation (x-axis) and the Radius of Gyration (y-axis) in nm calculated in the five concentrations of the crowded system (from 175 g/L to 296 g/L.) and for the CROWDED 4x (182 g/L and four times bigger). (TIF) [file pcbi.1005040.s005.tif]

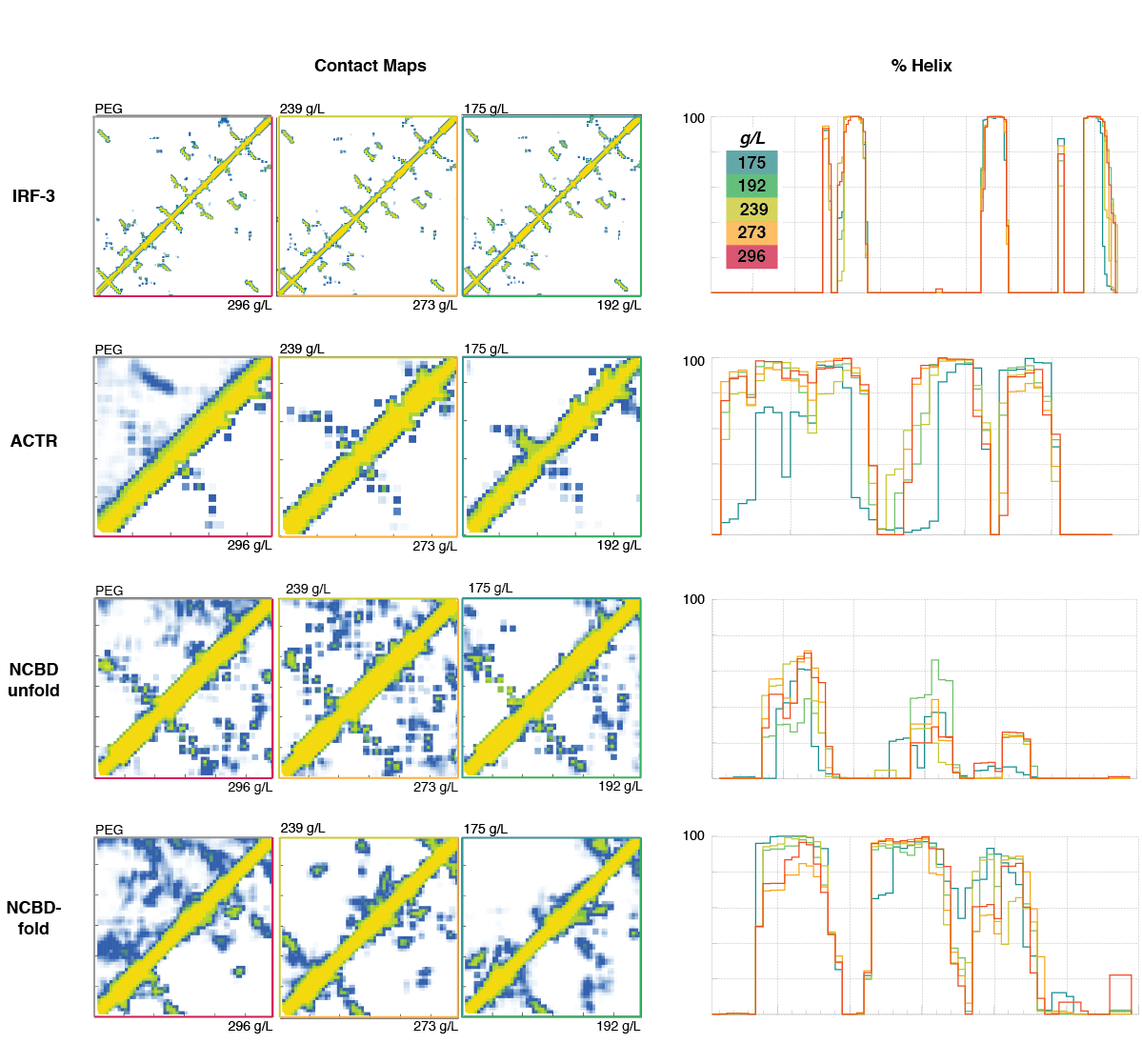

Supplement: S6 Fig — From the left: contact maps and the percentage of helixes along the protein sequence. In the case of NCBD, values are averaged for the three conformations (folded and unfolded). Color code as in Fig 1: gray for PEG500; dark green to red for crowding concentration from 175 g/L to 296 g/L. (PNG) [file pcbi.1005040.s006.png]

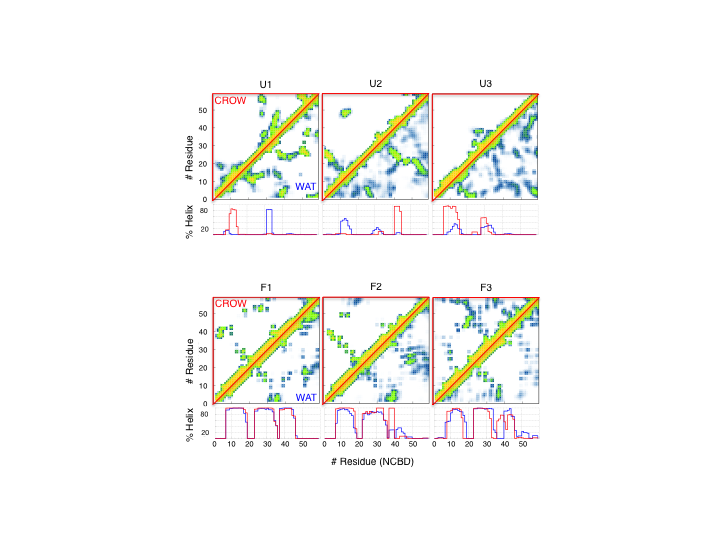

Supplement: S7 Fig — For each conformation (U1-3 and F1-3) the contact map and the percentage of helixes along the sequence in a crowded environment (192 g/L—red) and in water (blue). (TIFF) [file pcbi.1005040.s007.tiff]

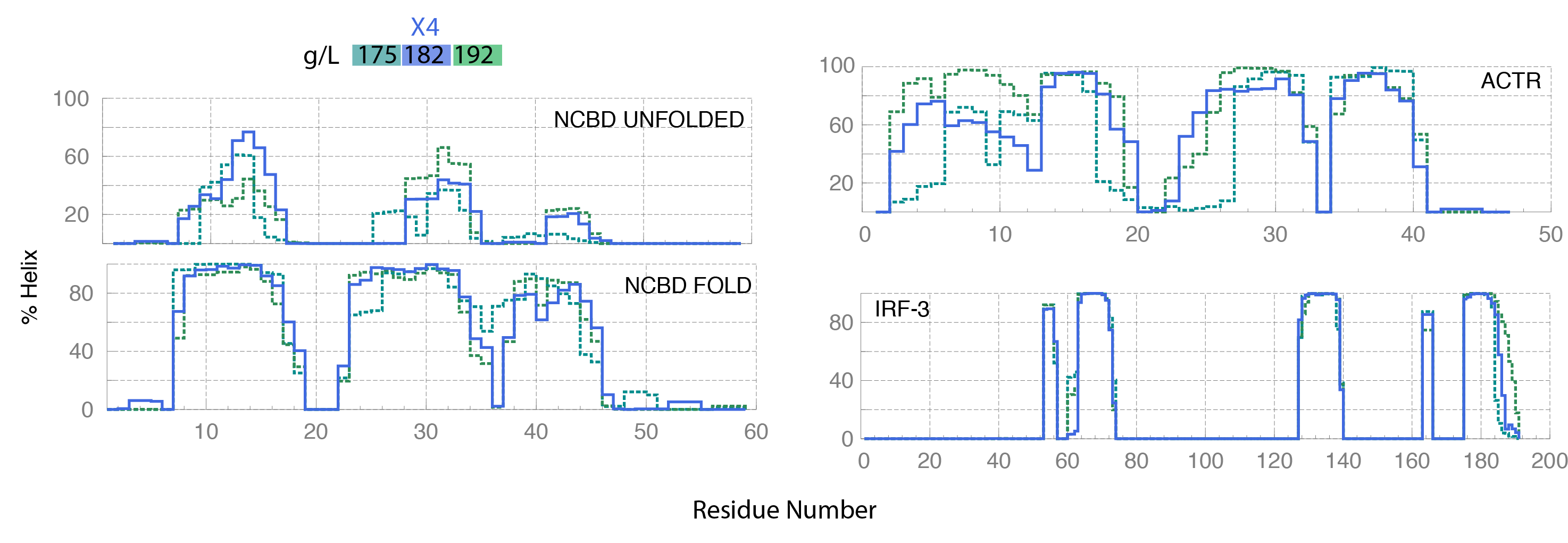

Supplement: S8 Fig — For each protein we compared the helical content calculated in all the conformations in the CROW 4X box (182 g/L) with the values taken from the crowding systems with comparable crowding concentration. (TIF) [file pcbi.1005040.s008.tif]

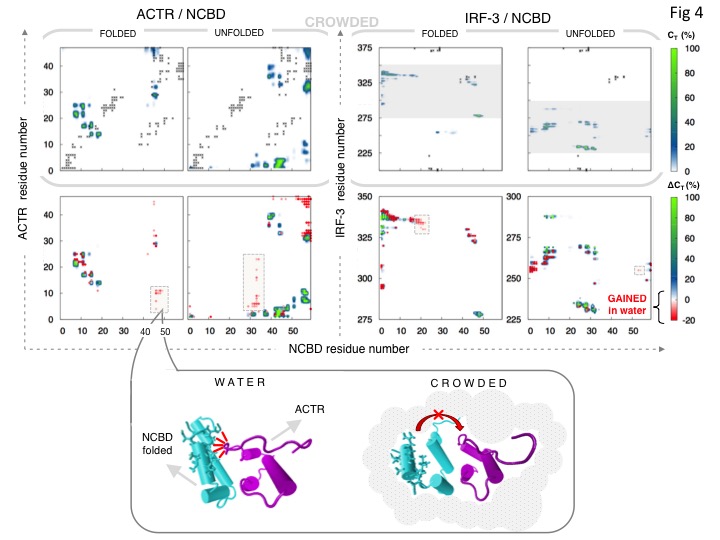

Supplement: S9 Fig — Contact maps between NCBD residues (x-axes) and its two partners (y-axes—ACTR on the left and IRF-3 on the right). The plots in the first row display the contact time (% of the total simulated time) in the simulation at 273 g/L as an example of a crowded system. The black dots mark contacts in the bounded structure available at the PDB. The second row displays the difference (Water—Crowded) in contact time calculated in the 10 copies of 10 ns in crowded conditions and in water. The contact map calculated from the 10 copies at crowded conditions is plotted in the background to identify contacts gained from scratch in water. The latter are marked with grey boxes. The cartoons at the bottom illustrate contacts newly formed in water (left side) between ACTR (in magenta) and NCBD (in cyan) while the crowded environment (right-side) prevented their formation. (JPG) [file pcbi.1005040.s009.jpg]

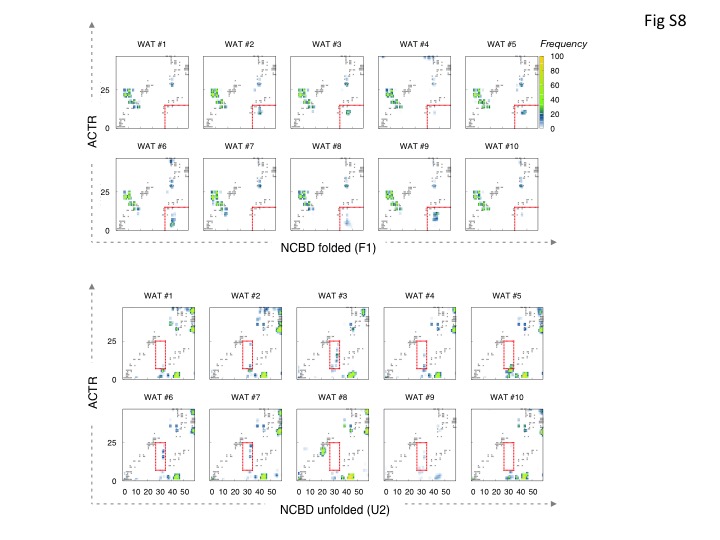

Supplement: S10 Fig — For each complex (ACTR with F1 or U2) the contact maps for each of the 10 copies in water are shown. The red boxes highlight areas where new contacts (not present in the crowded environment) are formed in water. (JPG) [file pcbi.1005040.s010.jpg]

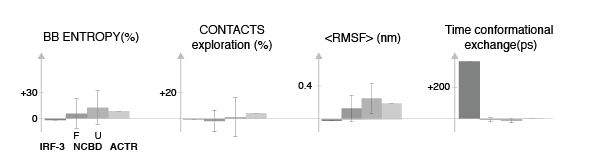

Supplement: S11 Fig — Starting from left: for each protein the % of intrinsic disorder (calculated with PONDR-FIT) and several differences using the simulation in water as reference: the backbone conformational entropy; the % of explored intra-protein contacts; the average local RMSF (Å) and the average time between conformational changes (ns). (PNG) [file pcbi.1005040.s011.png]
