# Supplementary material for: The Differential Response of Proteins to Macromolecular Crowding
Source: PLoS Comput Biol. 2016 Jul 29;12(7):e1005040. doi: 10.1371/journal.pcbi.1005040 (PMC4966950; doi:10.1371/journal.pcbi.1005040)
Supplement: S2 Table — The table reports the percentage of Lennard-Jones energies on the total calculated as the sum of Lennard-Jones and Coulomb energies between each protein and the rest of the system. (DOCX) [file pcbi.1005040.s013.docx]

| **Lennard –Jones term in the energy breakdown (%)*** | | | | |
| --- | --- | --- | --- | --- |
| **Protein** | **Conf.** | **Protein Crowding** | **PEG500** | **WATER** |
| **NCBD** | <avg> | 21.7 (1.6) | 20.4 (1.2) | 14.7 (0.8) |
|  | F1 | 19.7 (1.1) | 18.2 (1.2) | 14.5 (0.8) |
|  | F2 | 22.8 (1.5) | 19.4 (0.7) | 13.5 (0.7) |
|  | F3 | 19.9 (1.4) | 22.1 (1.4) | 15.6 (0.8) |
|  | U1 | 22.7 (1.9) | 20.2 (1.1) | 13.5 (0.9) |
|  | U2 | 23.4 (2.1) | 24.6 (2.2) | 14.7 (1.1) |
|  | U3 | 22.3 (1.9) | 21.5 (1.6) | 16.3 (1.1) |
| **ACTR** |  | 16.0 (1.0) | 12.5 (0.8) | 4.3 (0.3) |
| **IRF-3** |  | 13.2 (0.4) | 12.7 (0.5) | 12.4 (0.4) |

* % of the total given by the sum of the Lennard-Jones and the Coulombic energies calculated for each protein against the rest of the system.
