# Supplementary material for: The Differential Response of Proteins to Macromolecular Crowding
Source: PLoS Comput Biol. 2016 Jul 29;12(7):e1005040. doi: 10.1371/journal.pcbi.1005040 (PMC4966950; doi:10.1371/journal.pcbi.1005040)
Supplement: S3 Table — The table displays the average difference from simulation in water in several descriptors for each conformation of NCBD (Folded F1-3 and Unfolded U1-3) in presence of protein crowding (192 g/L) or PEG500. Values in bold are the average for each subgroup while the standard deviation is reported in brackets. (DOCX) [file pcbi.1005040.s014.docx]

|  | **% Helical** | | **BB conf.**  **entropy (%)** | | **Intra-Contacts**  **exploration (%)** | | **Time**  **Conform.**  **changes** | | **Average RMSF** | |
| --- | --- | --- | --- | --- | --- | --- | --- | --- | --- | --- |
|  | **CROW** | **PEG** | CROW | PEG | CROW | PEG | CROW | PEG | CROW | PEG |
| **Folded NCBD** | **2.64**  **(5.11)** | **1.33**  **3.01** | **-16.0**  **(11.1)** | **5.8**  **(16.8)** | **-32.6**  **(14.0)** | **-3.3**  **(15.6)** | **-37**  **(13)** | **-4**  **(10)** | **-0.24**  **(0.16)** | **0.13**  **(0.16)** |
| **Conf F1** | 1.13  (0.66) | -2.79 | -10.1  (13.2) | 22.1 | -20.5  (4.6) | 14.7 | -61  (27) | -10 | -0.17  (0.15) | 0.30  (0.16) |
| **Conf F2** | 10.34  (3.05) | 2.50 | -26.3  (6.7) | 6.7 | -50.2  (2.6) | -1.3 | -23  (12) | +1 | -0.39  (0.15) | 0.18  (0.16) |
| **Conf F3** | -3.40  (3.14) | 4.29 | -11.6  (4.1) | -11.5 | -27.0  (8.5) | -23.3 | -36  (12) | -3 | -0.15  ( 0.16) | -0.10  (0.16) |
| **Unfolded NCBD** | **5.15**  **(3.25)** | **12.72**  **5.34** | **-16.1**  **(15.5)** | **12.6**  **(19.1)** | **-57.1**  **(15.9)** | **2.5**  **(27.8)** | **+13**  **(23)** | **-10**  **(11)** | **-0.36**  **(0.17)** | **0.25**  **(0.18)** |
| **Conf U1** | 3.68  (3.03) | 9.75 | -1.4 | 34.5 | -36.4  (4.0) | 41.8 | +30 | -10 | 0.04  (0.15) | 0.68  (0.15) |
| **Conf U2** | 5.41  (3.51) | 20.22 | -26.0  (12.9) | 0.9 | -70.6  (6.8) | -19.0 | -12 | -16 | -0.51  (0.19) | 0.06  (0.20) |
| **Conf U3** | 6.36  (2.54) | 0.18 | -9.3  (0.6) | 2.3 | -27.0  (8.5) | -23.3 | +1 | -5 | -0.62  (0.17) | 0.00  (0.19) |
