# Supplementary material for: The Differential Response of Proteins to Macromolecular Crowding
Source: PLoS Comput Biol. 2016 Jul 29;12(7):e1005040. doi: 10.1371/journal.pcbi.1005040 (PMC4966950; doi:10.1371/journal.pcbi.1005040)
Supplement: Supplementary file 5 [file pcbi.1005040.s012.odt]

| Systems | Density (g/L) | Proteins inside the simulated box | Nº of simulations | Simulated time (per simulation) |
| --- | --- | --- | --- | --- |
| Protein Crowding | 175 | 1 ACTR : 6 NCBD (3 folded, 3 unfolded) : 1 IRF-3 | 1 | > 3 micro |
| 192 | 1 ACTR : 6 NCBD (3 folded, 3 unfolded) : 1 IRF-3 | 1 | > 3 micro |
| 239 | 1 ACTR : 6 NCBD (3 folded, 3 unfolded) : 1 IRF-3 | 1 | > 3 micro |
| 273 | 1 ACTR : 6 NCBD (3 folded, 3 unfolded) : 1 IRF-3 | 1 | > 3 micro |
| 296 | 1 ACTR : 6 NCBD (3 folded, 3 unfolded) : 1 IRF-3 | 1 | > 3 micro |
|  |  |  |  |  |
| PEG500 | 200 | Each protein alone | 8 | > 3 micro |
|  |  |  |  |  |
| Water | -- | Each protein alone | 8 | > 3 micro |
|  |  |  |  |  |
| Protein Crowding  4X | 183 | 4 ACTR : 24 NCBD (12 folded, 12 unfolded) : 4 IRF-3 | 1 | 100 ns |
|  |  |  |  |  |
| Binding partners in water | -- | 1 ACTR : 1 NCBD folded | 10 | 10 ns |
| -- | 1 ACTR : 1 NCBD unfolded | 10 | 10 ns |
| -- | 1 IRF-3 : 1 NCBD folded | 10 | 10 ns |
| -- | 1 IRF-3: 1 NCBD unfolded | 10 | 10 ns |
